# Electrospun Poly(acrylic acid-*co*-4-styrene sulfonate) as Potential Drug-Eluting Scaffolds for Targeted Chemotherapeutic Delivery Systems on Gastric (AGS) and Breast (MDA-Mb-231) Cancer Cell Lines

**DOI:** 10.3390/nano12213903

**Published:** 2022-11-04

**Authors:** Andrónico Neira-Carrillo, Ignacio A. Zárate, Eddie Nieto, Nicole Butto-Miranda, Lorena Lobos-González, Matias Del Campo-Smith, Daniel A. Palacio, Bruno F. Urbano

**Affiliations:** 1 Department of Biological and Animal Sciences, Faculty of Veterinary and Animal Sciences, University of Chile, Santa Rosa 11735, La Pintana, Santiago 8820808, Chile; 2 Advanced Center for Chronic Diseases (ACCDIS), Santiago 380492, Chile; 3 Center for Regenerative Medicine, Faculty of Medicine, Universidad del Desarrollo, Clínica Alemana, Santiago 7610658, Chile; 4 Department of Polymer Chemistry, Faculty of Chemical Science, University of Concepción, Concepción 3349001, Chile

**Keywords:** electrospun fibers, electrospinning, poly(acrylic acid-*co*-styrene sulfonate), gastric and breast cancer cells, clonogenic assay, polymers

## Abstract

Potential drug-eluting scaffolds of electrospun poly(acrylic acid-*co*-styrene sulfonate) P(AA-*co*-SS) in clonogenic assays using tumorigenic gastric and ovarian cancer cells were tested in vitro. Electrospun polymer nanofiber (EPnF) meshes of PAA and PSSNa homo- and P(AA-*co*-SS) copolymer composed of 30:70, 50:50, 70:30 acrylic acid (AA) and sodium 4-styrene sulfonate (SSNa) units were performed by electrospinning (ES). The synthesis, structural and morphological characterization of all EPnF meshes were analyzed by optical and electron microscopy (SEM-EDS), infrared spectroscopy (FTIR), contact angle, and X-ray diffraction (XRD) measurements. This study shows that different ratio of AA and SSNa of monomers in P(AA-*co*-SS) EPnF play a crucial role in clonogenic in vitro assays. We found that 50:50 P(AA-*co*-SS) EPnF mesh loaded with antineoplastic drugs can be an excellent suppressor of growth-independent anchored capacities in vitro assays and a good subcutaneous drug delivery system for chemotherapeutic medication in vivo model for surgical resection procedures in cancer research.

## 1. Introduction

Electrospinning (ES) is a simple and versatile fiber-spinning technology that uses an electric field to generate fibers at nano- and micro-scale by accelerating a jet of a charged polymer solution. Electrospun polymer nanofibers (EPnFs) meshes have enhanced and exceptional nanostructural characteristics, and they can be produced from either natural or synthetic biomedical polymers as well as composite and ceramic materials [1]. EPnFs such as fibers, medical threads, meshes, mats, and scaffolds are of utmost interest as advanced biomaterials with specific properties such as the high surface/volume ratio of a single filament, effective surface modification, special physicochemical behavior, among others [2]. Owing to its high porosity and large surface area, a non-woven mat of EPnFs can serve as an ideal scaffold to mimic the extracellular matrix (ECM) for cell attachment and nutrient transportation. EPnFs can provide a specific required architecture in biomedical applications. Replacing heart tissue or creating new blood vessels is possible by mimicking the three-dimensional ECM structure according to nanofiber fabrication methods and materials [3,4]. Therefore, EPnFs are a promising test-material platform for a range of biomedical applications that include controlled release, encapsulation, drug delivery, tissue engineering, and for diagnosis and therapy research [5]. Accordingly, the clinical use of nanofiber-based scaffolds remains challenging.

Chemotherapy, typically the main treatment of late-stage cancer or an adjunct method for surgery in early-stage cancer, suffers from systemic toxicity due to the unspecific biodistribution of chemotherapeutic drugs (chemo-drugs) for both cancerous and normal cells. Conventional chemotherapy is compromised by shortcomings and systemic toxicity. Consequently, it is urgent to achieve specific delivery to reduce side effects and optimize the therapeutic efficacy at lower doses [6]. It is well known that pharmacokinetics, distribution, and toxicity, among others, influence the administration form of a drug. Therefore, not only the drug is important when administering treatment. In this context, new “smart” biomaterials are always under investigation [7]. Thus, materials capable of responding to different stimuli have been described, such as magnetic, electric, thermal fields, etc. [8], where one of the most described being the use of pH-sensitive materials, which correspond to materials capable of releasing a molecule of interest depending on the pH of the environment [9]. In release studies, polymers that exhibit a stimulus or are sensitive to the medium are considered candidates for controlled release, where physiological stimulation by pH is one of the critical factors [10].

In the case of cancer, there are pH variations in the microenvironment, which can be used as a stimulus for drug release to occur [11,12]. It is in this sense, polymers containing acidic or basic chemical groups such as carboxylic acids and ammonium salts are sensitive to pH changes [10]. For example, poly(acrylic acid) (PAA) is a polyanion and is widely used in the formulation of drug release at neutral pH due to the ability to lose protons and thus acquire negative charges [10,13]. PAA is a low-cost, nontoxic, biocompatible, and bioadhesive polymer that requires no further treatment after insertion into the body. Thus, it is widely used in different applications such as superabsorbents, drug release, and water treatment [14,15,16]. On the other hand, polysodium 4-styrene sulfonate (PSS) is a synthetic polyelectrolyte with permanent negative charges and has been approved for food applications and drug administration. Different investigations have reported this polymer to possess biocompatibility, antimicrobial capacity, non-immunogenic activity, and non-cytotoxicity [17,18,19,20]. Polymers containing AA or SS monomer units are reported in applications as bone tissue scaffolds [21], osteogenesis in vitro [22], films for biomedical applications [23], and graft responses for synthetic ligaments [24]. On the other hand, the electrospinning process possesses numerous advantages that make it an ideal technique to be used in drug releases, such as high surface-to-volume ratio, high porosity, numerous routes of administration, easy fabrication, and control of fiber characteristics [25,26]. Although the use of PAA and PSS have been described in many applications, the preparation and characterization of copolymers with a precise proportion of AA and SS monomer units to be used, in the form of electrospun nanofibers, as a scaffold for drug delivery have not been reported.

The novelty of this work is the preparation of EPnFs meshes made of non-commercial homo- and copolymer materials with controlled monomer unit ratios and their evaluation as three-dimensional (3D) semisolid fibrillar scaffold of antineoplastic drugs for tumorigenic AGS and MDA-MB-231 cells instead of using a biopolymer material, e.g., polycaprolactone also used by us as 3D cell culture support for ovarian cancer cells (A2780). The current results could stimulate our group or others to test 3D EPnF meshes as scaffolds utilizing double anionic copolymer materials as a viable candidate for chemotherapeutic medication systems for in vivo subcutaneous delivery of clinical surgery [27].

In this study, AA and SSNa homo- and P(AA-*co*-SS) copolymer EPnF meshes with different monomer ratios were performed by using the ES technique. Herein, potential subcutaneous delivery of antineoplastic drugs using EPnF meshes for gastric (AGS) and breast cancer (MDA-Mb-231) cell lines in clonogenic in vitro assays are characterized and discussed as promising candidates in biomedical applications.

## 2. Materials and Methods

### 2.1. Reactants

Reagents of the highest available grade were used. All experiments and solution preparations were performed by using fresh Milli-Q distilled and bi-distilled water (LabostarTM TWF, Evoqua Water Technologies LLC, Warrendale, PA, USA). Sodium 4-styrene sulfonate and acrylic acid (99%) for the synthesis of P(AA-*co*-SS) copolymers and ethanol and dimethylformamide of analytical grade for the electrospun fiber meshes preparation was from Sigma-Aldrich purchased. Ammonium persulfate (98% APS) from Sigma-Aldrich was used as an initiator for the radical polymerization of P(AA-*co*-SS). Deuterium oxide (D_2_O) from Sigma-Aldrich. The omega-3 fatty acid docosahexaenoic acid (DHA) (Sigma Aldrich, St. Louis, Missouri, USA), cis-diamminedichloroplatinum (II) (CDDP) (Merck S.A., Darmstadt, Germany), and the papain (Pap) (Worthington Biochemical Corporation, Lakewood, NJ, USA) were utilized as chemotherapeutic/adjuvant drugs.

### 2.2. Synthesis of Homo- and Copolymers

The synthesis of PAA and PSSNa homo- and P(AA-*co*-SS) copolymers was carried out by free radical polymerization. Briefly, the AA monomer was purified by removing the 4-methoxy phenol used as a polymerization inhibitor in a packed column (Sigma-Aldrich, St. Louis, MO, USA). For the synthesis of the PAA and PSSNa homopolymers, 0.11 moles of the monomers were used, while the P(AA-*co*-SS) copolymers were synthesized by varying the molar ratio of AA:SS as follows 70:30; 50:50, and 30:70. The synthesis was conducted in aqueous solution with 1.0 mol% ammonium persulfate (APS) as a radical initiator with respect to the moles of the monomer in 40 mL of bi-distilled water under nitrogen (N_2_) gas atmosphere. All reactions were performed at 80 °C for 24 h. The purification process for the different polymers was carried out by dissolving them in approximately 2000 mL of water. Then, the polymers were fractionated using different membranes with molecular weight cutoffs of 10, 30, and 100 KDa. All purification studies were performed using deionized water at room temperature in the following order: first, the polymer solution was passed through membranes with a 10 kDa cutoff, the resulting solution greater than 10 kDa was fractionated with 30 kDa cutoff membranes, and finally, the polymer solution greater than 30 kDa is passed through 100 kDa membranes. Therefore, all synthesized polymers have molecular weights greater than or equal to 100 kDa.

### 2.3. Preparation of Electrospun Fiber Meshes by Electrospinning

Different solvents and processing parameters in the ES technique of homo- and copolymer P(AA-*co*-SS) EPnF meshes were utilized at room temperature. P(AA-*co*-SS) with 70:30, 50:50, and 30:70 AA:SS monomer ratio and homopolymers with random fiber orientation were prepared by using a flat plate collector. For PAA mesh preparation, an 8% (*w*/*v*) solution in a 1:1 water:ethanol mixture was utilized, and for the PSS mesh fabrication, a 20% (*w*/*v*) solution in a 2:1:1 dimethylformamide:water:ethanol mixture was used. For the 30:70 P(AA-*co*-SS) mesh preparation, a 25% (*w*/*v*) copolymer solution was prepared in a 2:1:1 mixture of dimethylformamide:water:ethanol. In the case of 70:30 and 50:50 P(AA-*co*-SS) meshes, 4% and 10% (*w*/*v*) copolymer solutions in 1:1 water:ethanol mixture were utilized. All polymer solutions were kept on an orbital shaker for 24 h, stirred on a magnetic stirrer at 25 °C for 2 h, and passed through a Swinnex holder (Millipore SigmaTM, Merck, Darmstadt, Germany) as standard protocol for preparing electrospun polymer fibers.

For the ES process 10 mL of homo- and copolymer solutions in a Nipro^®^ luer lock syringe was used and electrospun in an eStretching LE-10 Fluidnatek^®^ instrument. In general, all EPnF meshes were obtained at a constant room temperature of 25 °C using the following ES parameters: flow rate and voltage of 300–2200 µL/h and 9–18 kV, respectively. The distance between the collecting plate covered by aluminum foil and the metallic needle varied from 5 to 20 cm. In order to make the meshes comparable, 10 mL of the polymer solution was used for preparing each electrospun mesh.

### 2.4. Caracterización de EPnF Meshes

#### 2.4.1. Spectroscopic Characterization

P(AA-*co*-SS) copolymers with different comonomers ratios were analyzed by FT-IR and NMR. FT-IR analysis was performed on a FT-IR spectrometer (Nicolet Magna 550 spectrophotometer) (Nicolet Analytical Instruments, Madison, WI, USA). The FTIR spectra of all homo- and P(AA-*co*-SS) copolymer samples were recorded from 4000 cm^−1^ to 400 cm^−1^ at a resolution of 4 cm^−1^. The normalization procedure of FTIR spectra was performed by using absorption bands from monomeric units that did not change significantly as the internal standard for FTIR analysis of homo and copolymer P(AA-*co*-SS) samples. The ^1^H-NMR was obtained in an NMR spectrometer (Bruker Ascend 400 MHz) (Billerica, MA, USA). Deuterium oxide (D_2_O) was used as a deuterated solvent for NMR analysis.

#### 2.4.2. Contact Angle and Surface Tension of EPnF Meshes

Dulbecco’s modified Eagle medium (DMEM) was used to obtain the contact angle degree [°]. Measurements on all EPnF samples were performed by the Dataphysics optical contact system OCA 15EC (DataPhysics instruments GmBH, Filderstad, Germany). The dosing volume was 12 µL. The resulting average values are reported with standard deviation (±SD) after five replications for each sample. The surface tension of all EPnF meshes was obtained using the Young–Dupre equation (Equation (1)) [28,29]. For the surface tension determination of all samples, the surface tension of the DMEM liquid medium was previously determined by using Tate’s law (Equation (2)) [30,31]. In order to determine the mass of the drop, 30 drops of DMEM liquid were massed, and the average value was obtained. In order to minimize the hydrodynamic effect, the drops were obtained in a time-lapse of 30 s. Since the equation derived from Tate’s law assumes an ideal drop and that a high percentage of the drop’s mass remains in the capillary, a correction factor (Equation (3)) was added, which includes the correction factor *F* [31]. The calculation performed to determine the F parameter was taken from calculations made by Lee et al. (2009), as shown in Equation (4) [32].

(1) $$\sigma s=\sigma L\cdot \frac{{\left(1+\cos\theta \right)}^{2}}{4}$$

where,

*σL*: Surface tension DMEM
*σs*: Solid surface tension
*θ*: Contact angle between the surface of the solid and water

*m* · g = *γ* · 2 · π · *r* (2)

where:

*m*: Drop mass
g: Gravitational acceleration (9.8 m/s^2^)
*γ*: Surface tension at the droplet-air interface
2 · π · r: Wet perimeter

*m* · g = *F* · *γ* · 2 · π · *r* (3)

(4) $$F=1-0.9121\cdot \left(\frac{r}{V^{\frac{1}{3}}}\right)-2.109\cdot {\left(\frac{r}{V^{\frac{1}{3}}}\right)}^{2}+13.38\cdot {\left(\frac{r}{V^{\frac{1}{3}}}\right)}^{3}-27.29\cdot {\left(\frac{r}{V^{\frac{1}{3}}}\right)}^{4}+27.53\;\cdot {\left(\frac{r}{V^{\frac{1}{3}}}\right)}^{5}-13.58\cdot {\left(\frac{r}{V^{\frac{1}{3}}}\right)}^{6}+2.593\cdot {\left(\frac{r}{V^{\frac{1}{3}}}\right)}^{7}$$

#### 2.4.3. Morphological Analysis

Optical microscopy (OM) images were obtained in a Nikon Eclipse E400^®^ equipment and the LAZ program (Image Pro-Plus, Media Cybernetics, Melville, NY, UDA). The SEM-EDS analysis of the homo- and copolymer EPnF meshes was performed in a JEOL JSM-IT300LV microscope (JEOL USA Inc., Peabody, MA, USA), connected to an energy-dispersive X-ray detector for elemental microanalysis with computer-controlled Aztec EDX system software from Oxford Instruments, Abingdon, UK. The average diameter of electrospun fibers was determined by using ImageJ software (National Institute of Health, Bethesda, MD, USA). Three SEM images were used for each sample, and about 100 fibers were measured in each one, obtaining an *n* = 300 for each sample. The resulting average values of fibers were reported with standard deviation (±SD).

### 2.5. Biological Evaluation

#### 2.5.1. Cell Culture and Cell Viability

In general, for the cell culture and all biological evaluation, EPnF fiber meshes were sterilized by using 20 min exposure to ultraviolet light in a biosafety cabinet. Then the meshes were always loaded and handled under sterile conditions and in a cell culture cabinet. The human breast cancer cell line MDA-MB-231 (ATCC HTB-26) is an epithelial-like cell grown as a monolayer and in suspension in spinner cultures in DMEM-F12 (Gibco, Waltham, MA, USA). The human gastric cancer cell line AGS (ATCC CRL-1739) was derived from fragments of a tumor resected from a patient who did not receive prior therapy and grown as a monolayer in RPMI (Gibco). Cell culture media were supplemented with 10% fetal bovine serum (FBS, Hyclone, Logan, UT, USA), 10,000 U/mL penicillin, 10 mg/mL streptomycin sulfate and cells were cultured at 37 °C with 5% CO_2_ phase. On the other hand, after the EPnF meshes membrane were prepared, all were cut to the same size of 1 mm^2^. The manipulation of the EPnF meshes was performed with high-precision toothless needle-tipped dissecting forceps in such a way that only one tip of the membrane was grasped. Once cut, each of the meshes was placed in a culture plate, and different drugs were added to the center of the meshes, leaving them for a few minutes to adsorb and dry.

In this regard, well-known chemotherapeutic drugs described as selective inductors of apoptosis in tumor-derived cells and suppress tumor growth in gastric cancer, such as omega-3 fatty acid docosahexaenoic acid (DHA), cis-diamminedichloroplatinum (II) (CDDP), a platinum-based chemotherapy drug used to treat various types of cancers, and papain (Pap) described as a sulfhydryl proteases member of cathepsin family, useful as therapeutic targets for treating digestive cancers were utilized. Drugs were loaded by incorporating 1 µL to 5 µL volume of each drug in the center of the EPnF meshes as an adsorption process based on surface interactions between drugs and homo- and P(AA-*co*-SS) EPnF samples as the main drug-loading strategy. This process was repeated upon final verification that all loading volume was adsorbed. Since the platinum CDDP drug has an intense yellow coloration, it is very easy to monitor the adsorption on the polymeric meshes; a similar situation occurs with P1G10, which also has a yellow coloration, although much fainter. Finally, the cell viability assays of AGS cells were harvested at 24 h or 48 h post-culture in the presence of all EPnF meshes, and the total cell number and viability were determined by negative cells Trypan blue staining.

#### 2.5.2. Colony Formation Assay

Anchorage-independent cell growth was determined by colony formation in soft agar as previously described [33]. Briefly, AGS cells were seeded at 2 × 10^3^ live cells/well in 12-well plates in soft agar in the presence of EPnF meshes, in which always the same EPnF mesh size (1 mm^2^) was utilized. The formation of colonies > 100 µm in diameter was scored 3 weeks after seeding under a phase-contrast microscope at 10× The EPnF mesh that resulted more effective in the distribution of docosahexaenoic acid (DHA), CDDP, and papain was used in the same protocols with MDA-MB-231 breast cancer cells.

## 3. Results and Discussion

### 3.1. Spectroscopic Characterization of PAA, PSS Homopolymers, and P(AA-co-SS) Copolymers

**Figure 1:**
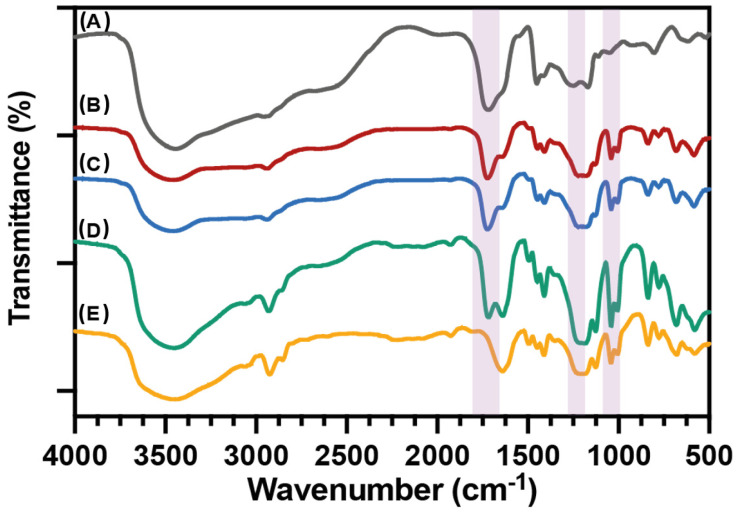
FTIR spectra of PAA and PSS homo- and P(AA-*co*-SS) copolymers. (**A**) PAA, (**B**) P(AA-*co*-SS) (70:30), (**C**) P(AA-*co*-SS) (50:50), (**D**) P(AA-*co*-SS) (30:70), (**E**) PSSNa.

Figure 1 shows the FTIR spectra of PAA and PSS homo- and P(AA-*co*-SS) copolymer samples. The spectrum of PAA displays absorption bands at 3242 cm^−1^ (stretching, O–H), 2930 cm^−1^ (C–H stretching), 1745 cm^−1^ (C=O stretching), 1461 cm^−1^ (C–O stretching), and 1187 cm^−1^ (CH_2_ rocking). While the FTIR spectrum of PSS displays the absorption bands at 2940 cm^−1^ (C–H stretching), 1660 cm^−1^ (aromatic C–C in-plane stretching), 1510 cm^−1^ and 1463 cm^−1^ (aromatic C–H in-plane stretching), 1423 cm^−1^ (CH_2_ bending), 1220 cm^−1^ (S=O stretching), and 1140 cm^−1^ (O=S=O stretching). P(AA-*co*-SS) copolymers display the characteristic bands of both monomer units, and their relative intensity varies with the composition, especially with the bands associated with C=O and S=O stretching [34].

**Figure 2:**
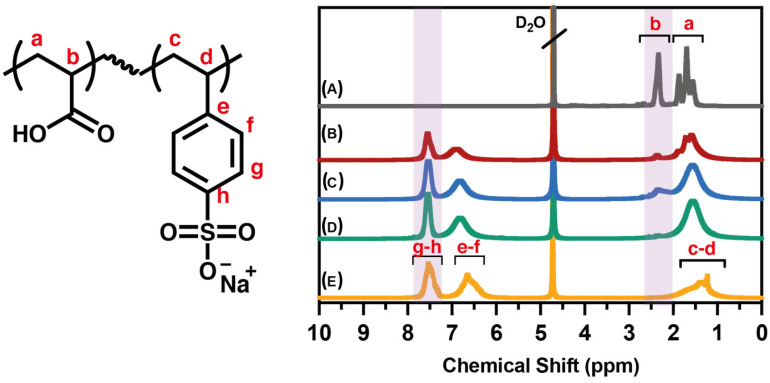
^1^H -NMR spectra of PAA and PSS homo- and P(AA-*co*-SS) copolymers. (**A**) PAA, (**B**) P(AA-*co*-SS) (70:30), (**C**) P(AA-*co*-SS) (50:50), (**D**) P(AA-*co*-SS) (30:70), (**E**) PSSNa. Deuterium oxide (D_2_O) was used as a deuterated solvent. Small letters of a, b, c, d, e, f, g and h assigned in the copolymer structure and ^1^H-NMR spectra indicate aliphatic and aromatic P(AA-*co*-SS) protons.

PAA and PSSNa homo- and P(AA-*co*-SS) copolymers were also characterized by ^1^H-NMR to verify their structures (Figure 2). The NMR analysis resulted in a strong analytical method to validate the chemical structure of all synthesized samples as well as to monitor the purity of homo- and P(AA-*co*-SS) copolymer samples by verifying that they are free from the starting monomer raw materials. In all NMR spectra, signals attributed to vinyl protons around 5.0 to 5.9 ppm are not observed, which indicates the absence of residual monomer as an impurity. In the ^1^H-NMR spectrum of PAA, signals between 1.1 and 2.1 ppm and 2.9–2.8 ppm PAA are assigned to –CH_2_ and –CH, respectively [35]. It is clarified that the protons of the carboxyl groups were not observed due to this chemical exchange process. The ^1^H-NMR spectrum of PSSNa displays broad signals between 6.5 ppm and 8.0 ppm, characteristic of aromatic protons, and in the range of 1.0 and 2.0 ppm ascribed to the protons of the polymer main chain [36]. However, ^1^H-NMR spectra of copolymers showed signals from both aromatic as well as aliphatic protons masked by the signals from PAA and PSSNa.

### 3.2. Characterization of EPnF Meshes

#### 3.2.1. Contact Angle and Surface Tension of EPnF Meshes

**Figure 3:**
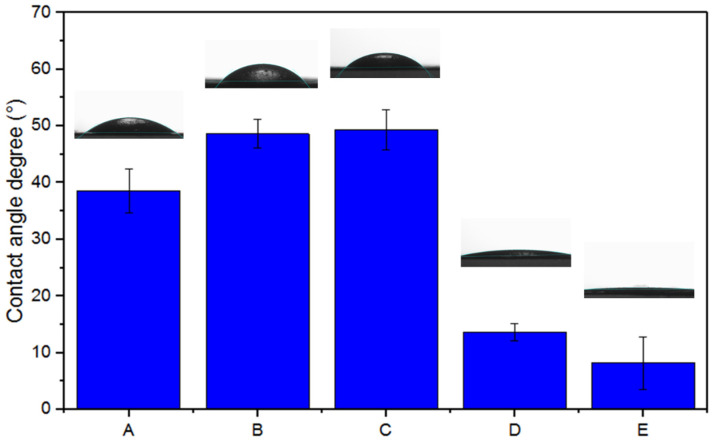
DMEM contact angle (°) measurements and surface optical images of homo- and copolymer EPnF meshes. (**A**) PAA, (**B**) P(AA-*co*-SS) (70:30), (**C**) P(AA-*co*-SS) (50:50), (**D**) P(AA-*co*-SS) (30:70) and (**E**) PSSNa.

DMEM liquid media provided in the clonogenic assays was utilized for all contact angle degree (°) measurements performed on EPnF mesh surfaces. Figure 3 shows the contact angle measurements and images at the surface of all EPnF meshes. The contact angle images of all copolymer EPnF after the spinning process showed that the surface organization of the continuous structure of fibers coherently corresponds to the wettability of AA and SSNa monomer units under the DMEM medium. We found that EPnF meshes obtained from PAA homopolymer had a contact angle of 39°. This result is consistent with the literature, which describes it as a hydrophilic material [37]. The PSSNa homopolymer mesh had a contact angle of 8°. In the case of copolymer fiber meshes, the P(AA-*co*-SS) (70:30) and P(AA-*co*-SS) (50:50) meshes had similar contact angles of 48° and 49°, respectively, while the P(AA-*co*-SS) (30:70) copolymer mesh, with a higher content of SS comonomeric unit, had similar behavior as PSSNa homopolymer with a contact angle of 13°. It is important to comment that all EPnF meshes have contact angle values less than 90° with good reproducibility. This experimental finding is a key factor when it is required to use some material with biomedical applications because it has been described that materials with hydrophilic characteristics promote adhesion to the extracellular matrix and cell biocompatibility [38,39,40].

Moreover, the surface tension (*σL*) of homo- and copolymers EPnF meshes in the presence of the DMEM medium can be quantitively determined using the Young–Dupre equation (Table 1), for which the interfacial surface tension between the surface of EPnF meshes and liquid medium DMEM was considered [28,29]. For this, the surface tension for the DMEM was experimentally determined as 69.3 using Tate’s law [30,31]. In order to achieve this, Equations (2)–(4) were previously used, and the surface tension of the DMEM was determined. The results are summarized in Table 2. With these data and using Equation (1), it was possible to calculate the surface tension of all EPnF meshes. Table 1 shows that the surface tension values are inversely related to the contact angle. Therefore, the highest surface tension values found for PSSNa homopolymer mesh (σL* = 68.6) and for P(AA-*co*-SS) (30:70) copolymer mesh (σL* = 67.3), with the highest content of SS unit, demonstrating that higher surface tension values indicate lower contact angle in our EPnF meshes (8° and 13°, respectively). The surface tension values determined in this study are consistent with those reported in the literature [41].

#### 3.2.2. Optical and Scanning Electron Microscopy

**Figure 4:**
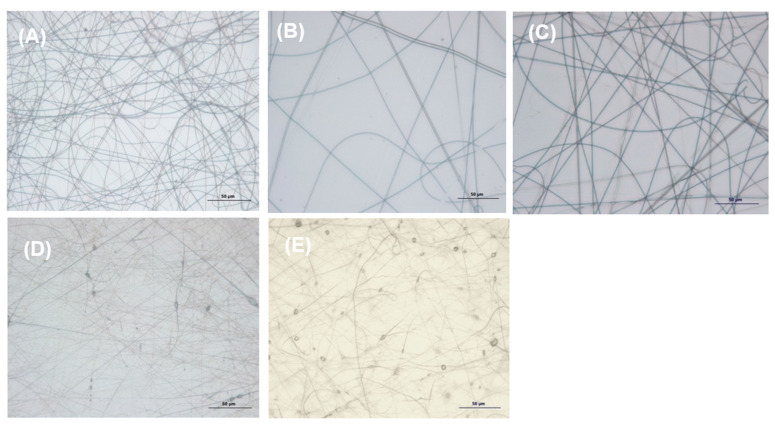
OM images illustrating random fibers of homo- and copolymer meshes using voltages of 18 kV at a distance of 20 cm. (**A**) PAA, (**B**) P(AA-*co*-SS) 70:30, (**C**) P(AA-*co*-SS) 50:50, (**D**) P(AA-*co*-SS) 30:70 and (**E**) PSS. Optical magnifications were 40×.

Using optical microscopy (OM), it was possible to preliminarily observe the presence of fibers and check their random fiber orientation of 50:50, 70:30, and 30:70 P(AA-*co*-SS) meshes (Figures S1–S3) and PAA and PSS fibers (Figure S4) using voltages from 9 to 18 kV and distance of 10–20 cm. The OM images presented in Figure 4 illustrate the EPnF fibers obtained at 18 kV at 20 cm on the aluminum foil substrate when the flat collector was used. Continuous EPnF meshes without morphological defects, in which dried P(AA-*co*-SS) copolymer fibers (Figure 4B–D) and PAA and PSS homopolymers (Figure 4A,E) at this voltage and distance were observed.

**Figure 5:**
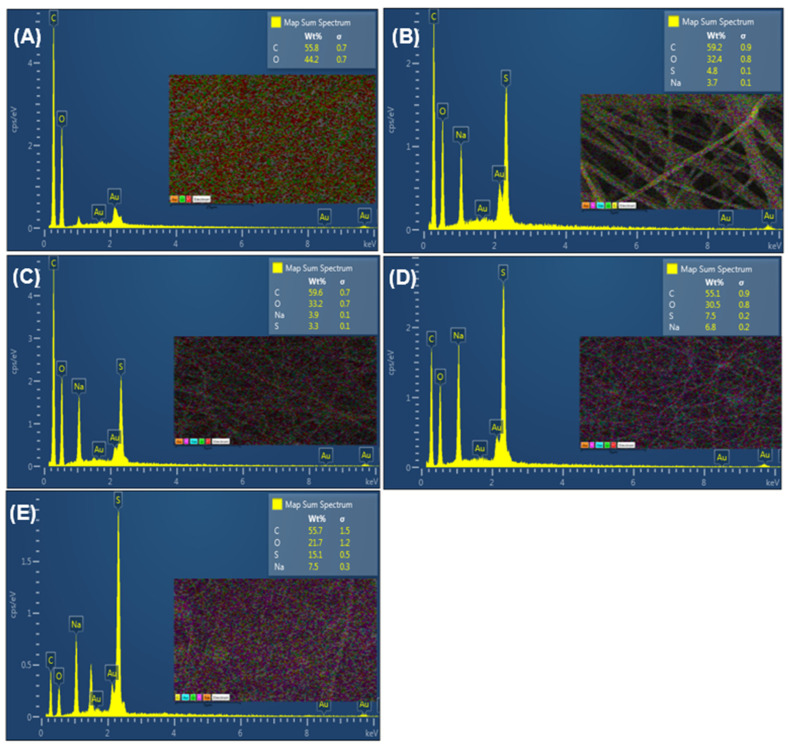
SEM-EDS EnPF meshes of homo- and PAA:PSS copolymers. (**A**) PAA, (**B**) P(AA-*co*-SS) (70:30), (**C**) P(AA-*co*-SS) (50:50), (**D**) P(AA-*co*-SS) (30:70), (**E**) PSSNa. The colored square represents the analyzed region on the sample of PAA. The colored square represents the analyzed region on the EPnF samples.

Surface morphology and microanalysis of EPnF meshes were studied by SEM-EDS analysis (Figure 5). EDS measurements were performed to determine the elemental composition of EPnF mesh. The composition of EPnF meshes and the percentages of each mesh’s elements were in agreement with the stoichiometry of PAA and PSSNa homo- and the copolymer EPnF samples. PAA fibers showed carbon and oxygen atoms, but they do not show the sulfur atom. However, for the mesh of PSSNa and all PAA:PSS copolymers always, the sulfur atoms were present, and as well as sodium atoms as they were neutralized and obtained as salts. Moreover, when the EDS measurements were performed on the PSSNa homopolymer, the content of sulfur atoms reached 15 wt%, while in the case of the copolymer EPnF mesh, the content of sulfur atoms was in the range of 7.5–3.3 wt%, as can be seen in Table 3 and Figure 5.

**Figure 6:**
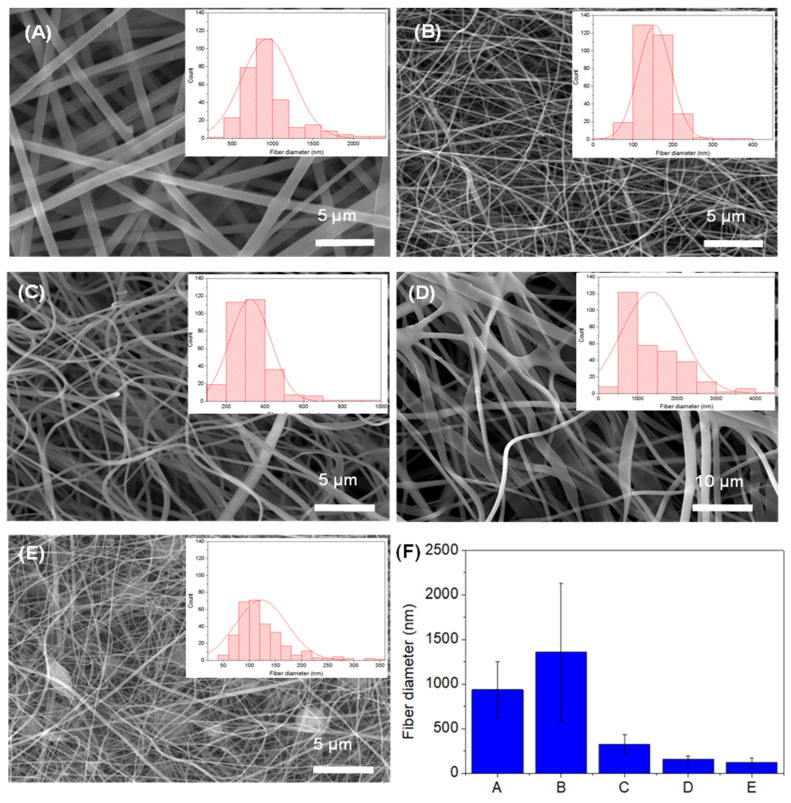
SEM images of PAA and PSSNa homopolymers and PAA:PSSNa copolymers EPnF meshes and the histograms representing the distribution of the fiber diameters. (**A**) PAA, (**B**) P(AA-*co*-SS) (70:30), (**C**) P(AA-*co*-SS) (50:50), (**D**) P(AA-*co*-SS) (30:70), (**E**) PSSNa and (**F**) Fiber size distribution graphs in all EPnF samples. The capital letters of A, B, C, D and E in the subfigure (F) indicate PAA, P(AA-*co*-SS) 70:30, P(AA-*co*-SS) 50:50, P(AA-*co*-SS) 30:70 and PSSNa, respectively.

Representative SEM images of EPnF samples show homogenous and free of bead formation on the surfaces (Figure 6). The copolymer EPnF meshes obtained with different PAA:PSSNa proportions show nanofibers without major morphological defects. They do not have a wet appearance and a flattened shape compared to fibers obtained from PAA and PSSNa polymers. The more flattering fiber appearance of PAA homopolymer mesh showed average fiber diameters of 1000 nm, and the PSSNa homopolymer fiber diameter is 140 nm. Our results showed that the P(AA-*co*-SS) 70:30 copolymer EPnF mesh has an average fiber diameter of 1000–1200 nm, while the P(AA-*co*-SS) 30:70 and P (AA-*co*-SS) 50:50 copolymers fiber diameter is 300 nm and 150 nm, respectively. These results show that the unit ratio is crucial, and the high content of AA or SS unit in P(AA-*co*-SS) copolymer mesh show a similar fiber diameter as of the respective homopolymers. We believe that the fiber diameter changes according to the coexistence of an anionic charge unit at the polymer solutions and its concentration during the formation of EPnF meshes during the electrospinning process. It is also important to comment that the occasional beads observed in PSSNa mesh completely disappear in all copolymer EPnF meshes, as it was demonstrated when different voltages and distances were used in the ES process as was described in the experimental section and observed by optical microscopy.

### 3.3. Biological Evaluation

#### 3.3.1. Viability Assay in Gastric Cancer Cells Assays of EPnF Meshes

**Figure 7:**
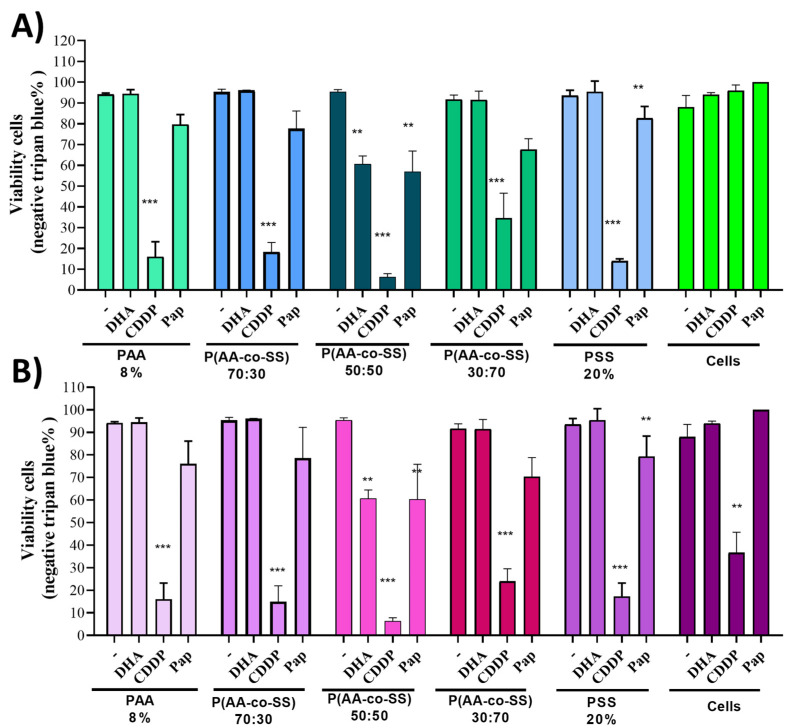
Cell viability of gastric tumoral cells AGS and breast cancer metastatic MDA-MB-231 in the presence of EPnF meshes charged with drugs. The cells (**A**) AGS tumoral gastric and (**B**) MDA-MB-231 breast cancer cells, were harvested in plate pre incorporated the different drugs charged meshes. After 24 or 48 h post-harvested, the total cell number and viability were determined by Trypan blue (Tb) as previously described [33,45]. ** *p* ˂ 0.001, *** *p* ˂ 0.0001.

In order to know toxicity effect on electrospun polymer fibers and if they can release molecules with some oxidative power that is capable of killing living cells, a viability test on the gastric cancer tumor cells AGS was performed. The cells were seeded and grown in culture plates for 48 h in the presence of EPnF meshes that were in suspension, those that degraded after 48 h making them not visible, however, the cells only died when the EPnF meshes were loaded with a classic chemotherapeutic as cisplatin (Figure 7). This result demonstrated that the proliferative property of the tested cells was not affected in the presence of EPnF meshes using different loads compared to the control cells, which have only the wild-type polymeric matrix.

Although the full understanding of the interaction of EPnF meshes with drugs is crucial, however, this was not specifically addressed in this study. According to the drug adsorption loading methodology and the type of electrospun fiber meshes, the loading system, such as coaxial and blend solution protocol between drug and EPnF meshes, is discarded [42,43]. With this in mind, we believe that interactions between double anionic functional groups of EPnF meshes with drugs occur at the surface as a principal mechanism due to the fact that the drug solution was applied on meshes after the electrospinning process. Considering the functional chemical properties of homo- and P(AA-*co*-SS) copolymer samples and the nature of drugs, the release mechanism is governed by the surface interactions as well as the drug solubility properties [44].

**Figure 8:**
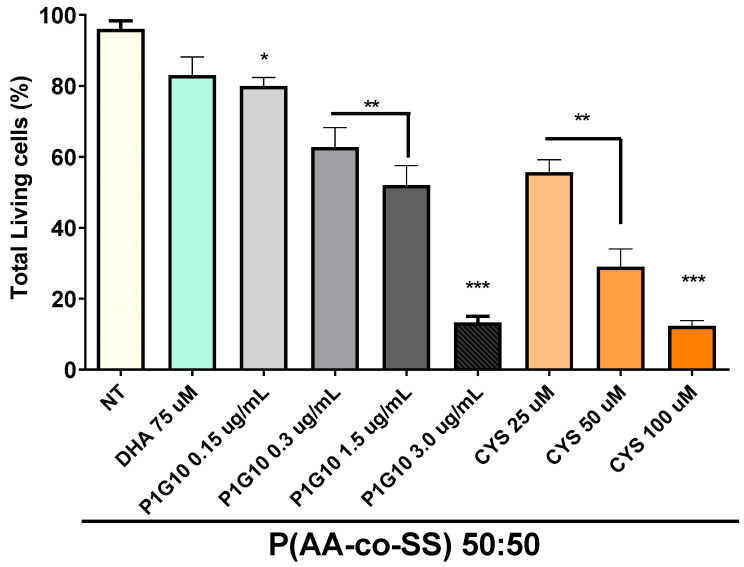
The proliferation rate of human hES cells in the presence of 50:50 P (AA:SS) copolymer mesh charged with 20 µL of CDDP drug. 5 × 10^3^ of hES cells were seeded in the center of the mesh, and the proliferation rate was evaluated at 24 h. The graph shows the total living number of hES cells cultured in CDDP at 25 µM, 50 µM and 100 µM, P1G10 at 0.15 µg/mL, 0.3 µg/mL, 1.5 µg/mL and, 3.0 µg/mL and left untreated (NT). Ordinary one-way analysis of variance ANOVA with respect to NT (* *p* ˂ 0.01, ** *p* ˂ 0.001, *** *p* ˂ 0.0001) was used.

The EPnF meshes were charged with different compounds similar to Ω-3 fatty acid docosahexaenoic acid (DHA). These were recently described as selective inductors of apoptosis in tumor-derived cells and suppress tumor growth in gastric cancer [46]. Cisplatinum, or cis-diamminedichloroplatinum (II), is a well-known chemotherapeutic drug with different abbreviation names. In this study, the current chemotherapy medication is designated as CDDP [47]. Papain (Pap) is a proteases member of the cathepsin family. Due to their important roles in digestive tumors, cathepsins might be therapeutic targets for treating digestive cancers [48,49]. The concentration of drugs used was DHA 50 µM, CDDP 100 µM, and Pap 1.5 µg/mL, respectively. The results show how CDDP is distributed adequately and decreases the tumoral cell viability in all EPnF meshes. Surprisingly, the 50:50 P(AA-*co*-SS) had the capability to reduce tumoral cell viability when charged with DHA and Pap drugs, while the other stoichiometry of copolymer EPnF meshes did not show the same tumorigenic behavior. For this reason, the clonogenic assays could be the response from other capacities of the tumoral cells, e.g., the growth of independent anchored. In order to show the effect of EPnF meshes with cisplatin on healthy cell lines, the proliferation rate of human gastric epithelial stem cells (hES) on 50:50 P(AA-*co*-SS) copolymer mesh using CDDP at different concentrations with 20 µL of loading volume was evaluated (Figure 8). These results suggest that the EPnF mesh did not significantly affect the hES cell viability. The adsorption of the total loading volume was performed by 2 µL to 2 µL, where a drying step was required in between.

#### 3.3.2. Clonogenic Assay in Gastric and Breast Cancer Cells Using EPnF Meshes

**Figure 9:**
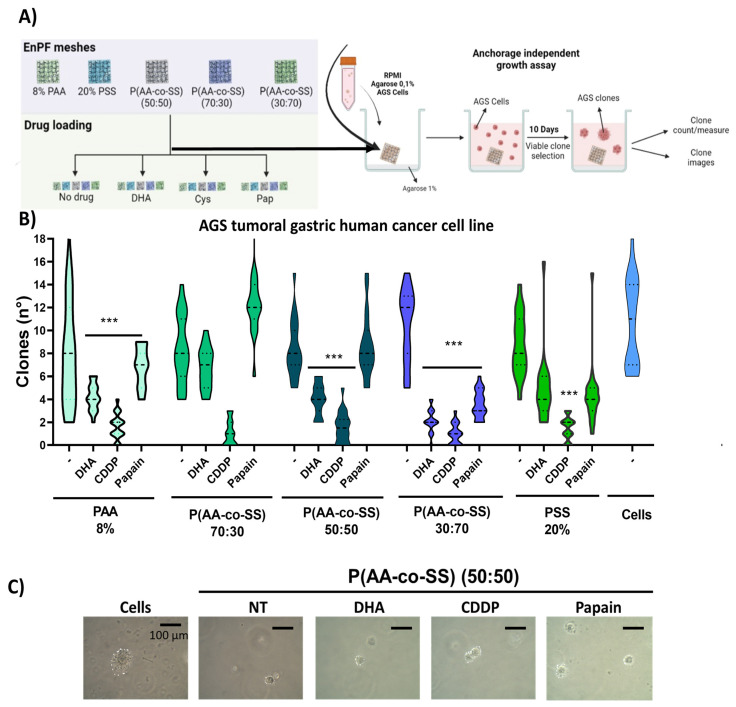
Anchored-independent cell growth assay of gastric AGS tumoral cells in the presence of EPnF mesh charged with chemotherapeutic/adjuvant drugs. (**A**) Experimental design for evaluating the anchorage-independent growth ability of tumoral cells in each EPnF mesh. (**B**) The total number of clones at 10 days, in which AGS cells cultured with 8% PAA, P(AA-*co*-SS) (70:30), P(AA-*co*-SS) (50:50), P(AA-*co*-SS) (30:70), and 20% PSS. EPnF meshes were charged with 50 µM of DHA, 100 µM of CDDP, and 1.5 µg/mL of Pap. The graph showed the total number of clones formed and representative optical images from clonogenic assays under each condition. The amplification bar represents 100 µm (*n* = 3, *** *p* ˂ 0.001), (**C**) representative AGS tumoral cells are growing clones in the soft agar.

**Figure 10:**
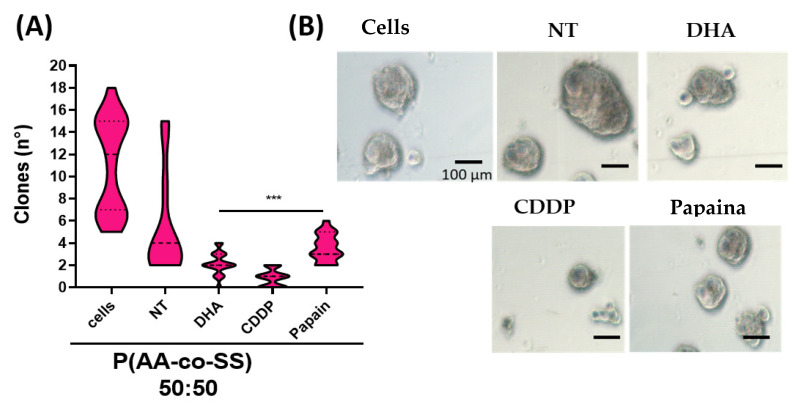
Growth independent anchored assays of MDA-Mb-231 breast cancer cells in the presence of EpnF mesh charged with chemotherapeutic/adjuvant drugs. (**A**) The breast cancer cells MDA-MB-231 were cultured in the absence of cells and with P(AA-*co*-SS) (50:50) loaded with 50 µM of DHA, 100 µM of CDDP, and 1.5 µg/mL Pap in semi-solid agar. The graph showed the total number of clones formed and representative optical images from clonogenic assays under each condition. The amplification bar represents 100 µm (*n* = 3, *** *p* ˂ 0.001). (**B**) OM images from clone growing assays with P(AA-*co*-SS) (50:50) mesh. The amplification bar is 100 µm in the MDA-Mb-231 gastric cancer using P(AA-*co*-SS) (50:50) and charged with 50 µM of DHA, 100 µM of CDDP, and 1.5 µg/mL of Pap.

DMEM, as mentioned, cell viability is not affected by cell proliferation. This occurs only when the EPnF samples were loaded with CDDP. However, considering future exploration using EPnF meshes, we evaluate the potential cell capacity to grow independent of the anchor. Although this property is a malignant characteristic of tumor cells, it is also a necessary characteristic for wound healing. With this in mind, we evaluated the anchorage-independent growth ability of the gastric cancer AGS cells and breast cancer MDA-MB-231 cells. Clonogenic assays of gastric and breast cancer cells in the presence of EPnF mesh charged with chemotherapeutic/adjuvant drugs were explored (Figure 9 and Figure 10). The AGS tumoral cells were seeded in soft agar semisolid with EPnF meshes in the middle of the agar. The 1 × 10^5^ cells were seeding mixed with semi-solid agar at 3% in RPMI medium. At 10 days, the colony was counted and measured. Our results show that unloaded EPnF meshes do not affect the growing cell capacity, suggesting that they can be projected for use in wound closures or for breast reconstructions in mastectomies in case of breast cancer. Surprisingly we found that when the EPnF meshes were loaded with chemotherapy. This capacity decreased at least eight times in all cases (Figure 9). The growth-independent capacity was measured in semisolid viability and growth model. By using the same design as the viability assays, the EPnF meshes were charged with DHA, CDDP, and Pap. Our findings show how CDDP was adequately distributed and decreased the tumoral cell growing independent capacities in all EPnF meshes (Figure 10). Indeed, 50:50 P(AA-*co*-SS) mesh had the major ability to suppress this capacity when it was charged with DHA and Pap drugs compared to 70:30 and 30:70 EPnF meshes. It is important to mention that EPnf meshes could be one of the new scaffold-type biomaterials to be used in the future for the local delivery of therapies [50], also being an excellent material to facilitate wound closures and new vasculature processes [51].

We highlight that the outcomes of this in vitro research represent a first approach to the study of the use of these electrospun polymeric materials in cancer-releasing common drugs (CDDP) as plant extracts (Papain), considering that these structures would be a viable platform type for localized drug delivery system [42,43,44], and in the future projecting its use in a tumor resection area in a patient where there may be angiogenesis [51]. It is for this reason that we must advance this research in a biosecure manner. We know that the current assays only evaluate the proliferative capacity of the cells and the capacity to grow independently of anchorage, which is an important tumorigenic capacity in tumor recurrence [52]. It is also very important to mention that scaffolds have been described for the delivery of stem cells to tissues that require regeneration [53,54,55]. However, it is key to consider the risk of delivering totipotent cells in a tumor environment since the cells that are intended to regenerate the resected tissue may end up being tumorigenic and metastatic cells. For this reason, many of the clinical trials in which stem cells and tissue regeneration were combined have not been successful because of the large generation of teratomas.

## 4. Conclusions

P(AA-*co*-SS) samples with different AA:SSNa monomeric ratios were synthesized and confirmed by FTIR and ^1^HNMR analysis. Continuous EPnF meshes without morphological defects were prepared, and their morphology was demonstrated by SEM-EDS. Clonogenic assays in gastric and breast cancer cell lines show that P(AA-*co*-SS) meshes with 50:50 AA:SSNa monomers ratio represent a promising candidate for targeted chemotherapeutic delivery systems, suggesting here by using a semisolid scaffold of antineoplastic drugs for tumorigenic AGS and MDA-MB-231 cells. In summary, EPnF meshes of P(AA-*co*-SS) as 3D structural fibrillar matrices deserve further investigation for biomedical applications.

## Figures and Tables

**Table 1:** Surface tension values of all EnPF meshes.

Surface Tension *σL*	PAA	PAA:PSS (70:30)	PAA:PSS 50:50)	PAA:PSS (30:70)	PSSNa
*σL* ^a^	52.3	45.9	44.6	66.9	67.5
*σL* *	55.0	47.8	47.2	67.3	68.6
*σL* ^b^	57.5	49.6	49.8	67.7	69.1

The ^a^ and ^b^ letters and the asterisk (*) on the surface tension (*σL*) indicate the maximum, minimum, and average surface tension values of the EPnF meshes, respectively.

**Table 2:** Data used for determination of surface tension of the DMEM liquid media.

Radio (m)	Density (kg/m^3^)	Drop Mass (kg)	Drop Volume (m^3^)	$\frac{r}{V^{\frac{1}{3}}}$	F	Surface Tension DMEM
0.00194641	1001	5.61 × 10^−5^	5.60 × 10^−8^	0.5096	0.649196026	69.28

**Table 3:** EDS measurements on EnPF meshes of PAA and PSSNa homopolymers and PAA:PSS copolymers.

Sample	%C	%O	%S	%Na	Total wt%
PAA	55.8	44.2	-	-	100
PAA:PSS (70:30)	59.2	32.4	4.8	3.7	100
PAA:PSS (50:50)	59.6	33.2	3.3	3.9	100
PAA:PSS (30:70)	55.1	30.5	7.5	6.8	100
PSSNa	55.7	21.7	15.1	7.5	100

C, O, S, and Na are carbon, oxygen, sulfur, and sodium atoms in EnPF meshes. The script line indicates that the atom is absent in the sample.

## Data Availability

The data will be made available on request.

## Supplementary Materials

The following are available online at https://www.mdpi.com/article/10.3390/nano12213903/s1, Figure S1: MO images of random 25% P(AA-*co*-SS) 30:70 fibers obtained by using voltages of 9–18 and at a distance of 10–20 cm, Figure S2: MO images of random 10% P(AA-*co*-SS) 50:50 fibers obtained by using voltages of 9–18 and at a distance of 10–20 cm, Figure S3: MO images of random 10% P(AA-*co*-SS) 70:30 fibers obtained by using voltages of 9–18 and at a distance of 10–20 cm, Figure: S4 MO images of random fibers from 8% PAA and 20% PSSNa using voltages of 12–18 and at a distance of 20 cm.
