# Supplementary material for: Electrospun Poly(acrylic acid-co-4-styrene sulfonate) as Potential Drug-Eluting Scaffolds for Targeted Chemotherapeutic Delivery Systems on Gastric (AGS) and Breast (MDA-Mb-231) Cancer Cell Lines
Source: Nanomaterials (Basel). 2022 Nov 4;12(21):3903. doi: 10.3390/nano12213903 (PMC9657868; doi:10.3390/nano12213903)
Supplement: Supplementary file 1 [file nanomaterials-12-03903-s001.zip › nanomaterials-1949033-supplementary revised version.pdf]

nanomaterials

Supplementary Materials

Electrospun Poly (Acrylic Acid-*co*-4-styrene Sulfonate) as Potential Drug-Eluting Scaffolds for Targeted Chemotherapeutic Delivery Systems on Gastric (AGS) and Breast (MDA-Mb-231) Cancer Cell Lines

Andrónico Neira-Carrillo [1,2,*], Ignacio A. Zárate [1], Eddie Nieto [1], Nicole Butto-Miranda [1], Lorena Lobos-González [2,3], Matias Del Campo-Smith [2,3], Daniel A. Palacio [4] and Bruno F. Urbano [4]

[1] Department of Biological and Animal Sciences, Faculty of Veterinary and Animal Sciences, University of Chile, Santa Rosa 11735, La Pintana, Santiago 8820808, Chile
[2] Advanced Center for Chronic Diseases (ACCDIS), Santiago 380492, Chile
[3] Center for Regenerative Medicine, Faculty of Medicine, Universidad del Desarrollo, Clínica Alemana, Santiago 7610658, Chile
[4] Department of Polymer Chemistry, Faculty of Chemical Science, University of Concepción, Concepción 3349001, Chile
* Correspondence: aneira@uchile.cl; Tel.: +56-22-978-5642

3. Supporting Results and Discussion Section

3.1. Characterization of EPnF meshes

Characterization of EPnF meshes by using Optical microscopy (OM). Using the optical microscopy (OM), it was possible to observe the presence and the fiber orientation of P(AA-co-SS) (Figures S1, S2 and S3), and PAA and PSSNa fibers (Figure S4) on the aluminum foil substrate when the flat collector was used, respectively. In both cases, continuous EPnF meshes without morphological defects was obtained by controlling the applied voltage and the distance as key ES parameters. For P(AA-co-SS) copolymer meshes using the three AA:SS monomer ratio, we observe continuous fine dried fibers at higher voltage (up to 12 kV) than EPnF homopolymer meshes at the distance from 5 to 20 cm, in which dried fibers of PAA and PSSNa meshes appeared from 9Kv but with distance from 15 to 20 cm (Figure S4A–F). For the preparation of 30:70 P(AA-co-SS) mesh, we observe only continuous dried fine fibers from 12 kV and distance from 5 to 20 cm. In general, non-continuous and wet fibers were obtained for the homopolymer meshes at low voltages (9 kV) and short distances from 5 to 10 cm, however, continuous dried fibers of PAA mesh were achieved when these ES parameters were higher than 9 kV with distances in the range of 15 to 20 cm (Figure S4A,B).

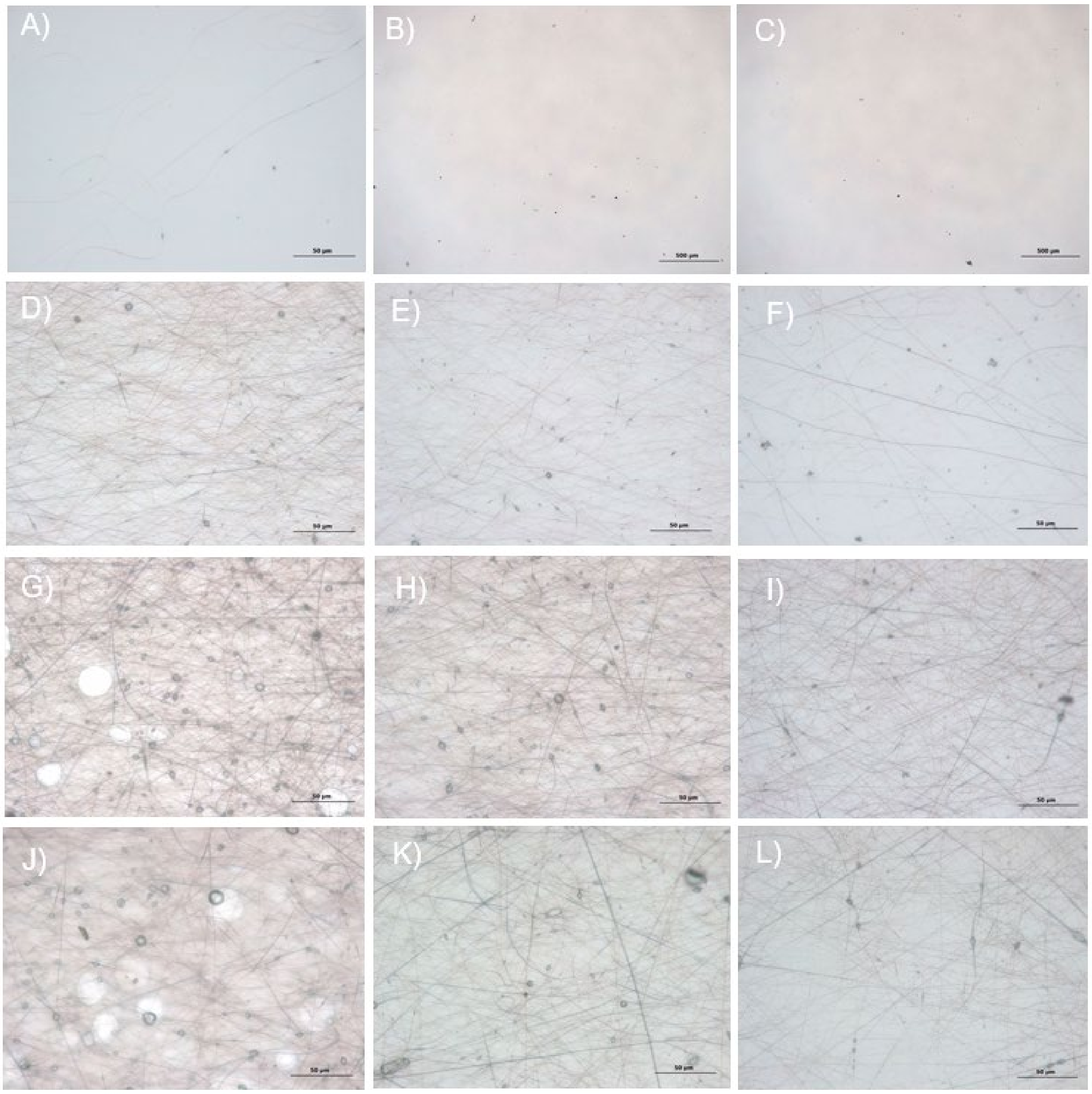

Figure S1. Optical microscopy images of random 25% 30:70 P(AA-*co*-SS) mesh obtained with voltages of 9-18 and at a distance of 10-20 cm: (**A**) P(AA-*co*-SS) fibers at 9 kV 10 cm, (**B**) P(AA-*co*-SS) fibers at 9 kV 15 cm, (**C**) P(AA-*co*-SS) fibers at 9 kV 20 cm, (**D**) P(AA-*co*-SS) fibers at 12 kV 10 cm, (**E**) P(AA-co-SS) fibers at 12 kV 15 cm, (**F**) P(AA-*co*-SS) fibers at 12 kV 20 cm, (**G**) P(AA-*co*-SS) fibers at 15 kV 10 cm, (**H**) P(AA-*co*-SS) fibers at 15 kV 15 cm, (**I**) P(AA-*co*-SS) fibers at 15 kV 20 cm, (**J**) P(AA-*co*-SS) fibers at 18 kV 10 cm, (**K**) P(AA-*co*-SS) fibers at 18 kV 15 cm and (**L**) P(AA-*co*-SS) fibers at 18 kV 20 cm. Optical magnifications were of 40×.

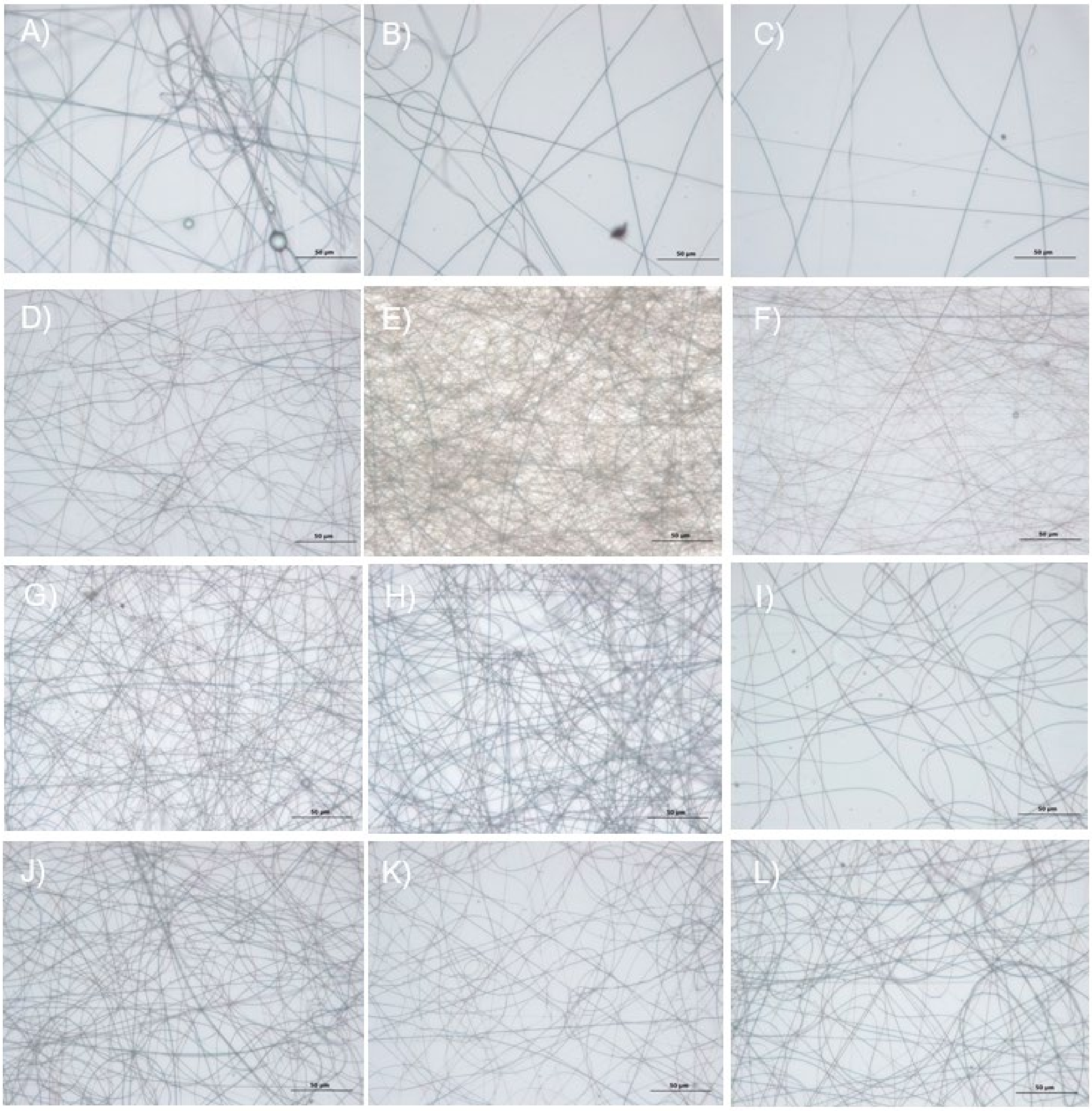

Figure S2. Optical microscopy images of random 10% 50:50 P(AA-*co*-SS) mesh obtained with voltages of 9-18 and at a distance of 10-20 cm: (**A**) P(AA-*co*-SS) fibers at 9 kV 10 cm, (**B**) P(AA-co-SS) fibers at 9 kV 15 cm, (**C**) P(AA-*co*-SS) fibers at 9 kV 20 cm, (**D**) P(AA-*co*-SS) fibers at 12 kV 10 cm, (**E**) P(AA-co-SS) fibers at 12 kV 15 cm, (**F**) P(AA-*co*-SS) fibers at 12 kV 20 cm, (**G**) P(AA-*co*-SS) fibers at 15 kV 10 cm, (**H**) P(AA-*co*-SS) fibers at 15 kV 15 cm, (**I**) P(AA-*co*-SS) fibers at 15 kV 20 cm, (**J**) P(AA-*co*-SS) fibers at 18 kV 10 cm, (**K**) P(AA-*co*-SS) fibers at 18 kV 15 cm and (**L**) P(AA-*co*-SS) fibers at 18 kV 20 cm. Optical magnifications were of 40×.

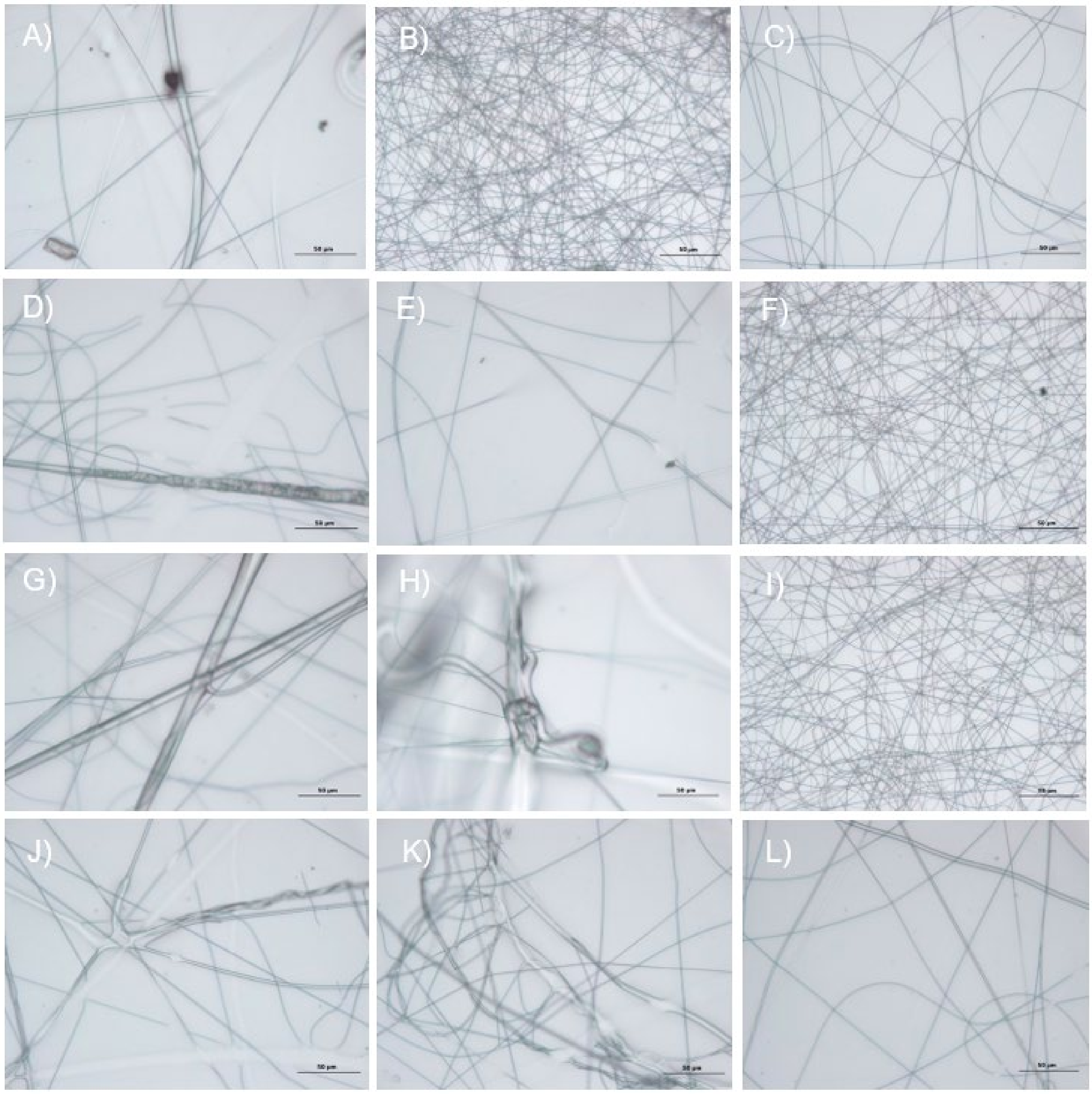

Figure S3. Optical microscopy images of random 4% 70:30 P(AA-co-SS) mesh obtained with voltages of 9-18 and at a distance of 10-20 cm: (**A**) P(AA-co-SS) fibers at 9 kV 10 cm, (**B**) P(AA-*co*-SS) fibers at 9 kV 15 cm, (**C**) P(AA-co-SS) fibers at 9 kV 20 cm, (**D**) P(AA-*co*-SS) fibers at 12 kV 10 cm, (**E**) P(AA-co-SS) fibers at 12 kV 15 cm, (**F**) P(AA-*co*-SS) fibers at 12 kV 20 cm, (**G**) P(AA-*co*-SS) fibers at 15 kV 10 cm, (**H**) P(AA-*co*-SS) fibers at 15 kV 15 cm, (**I**) P(AA-*co*-SS) fibers at 15 kV 20 cm, (**J**) P(AA-*co*-SS) fibers at 18 kV 10 cm, (**K**) P(AA-*co*-SS) fibers at 18 kV 15 cm and (**L**) P(AA-*co*-SS) fibers at 18 kV 20 cm. Optical magnifications were of 40×.

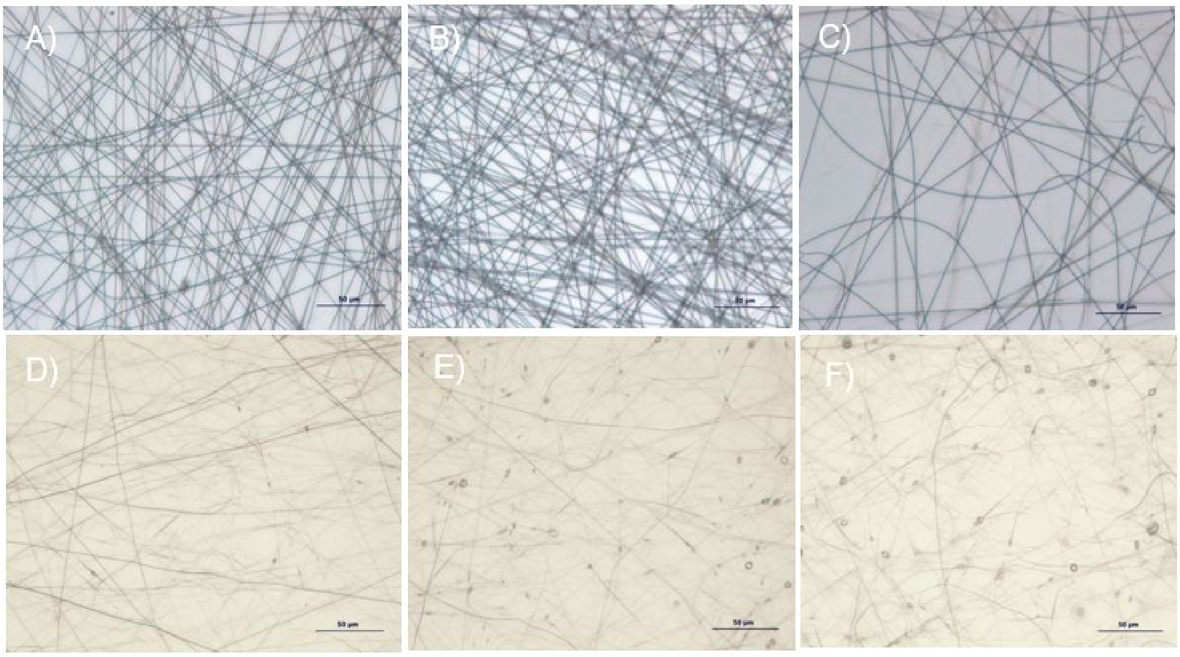

Figure S4. Optical microscopy images showing random fibers from 8% PAA and 20% PSSNa using voltages of 12 -18 and at a distance of 20 cm: (**A**) PAA fibers at 12 kV, (**B**) PAA fibers at 15 kV, (**C**) PAA fibers at 18 kV, (**D**) PSS fibers at 12 kV, (**E**) PSS fibers at 15 kV and (**F**) PSS fibers at 18 kV. Optical magnifications were of 40×.
